# Relationship between pre-anesthetic and intra-anesthetic airway resistance in patients undergoing general anesthesia: A prospective observational study

**DOI:** 10.1371/journal.pone.0172421

**Published:** 2017-02-17

**Authors:** Takamitsu Ikeda, Kanji Uchida, Yasuhiro Yamauchi, Takahide Nagase, Koji Oba, Yoshitsugu Yamada

**Affiliations:** 1 Department of Anesthesiology, Graduate School of Medicine, The University of Tokyo, Tokyo, Japan; 2 Department of Respiratory Medicine, Graduate School of Medicine, The University of Tokyo, Tokyo, Japan; 3 Department of Biostatistics, School of Public Health, Graduate School of Medicine, The University of Tokyo, Tokyo, Japan; Southeast University Zhongda Hospital, CHINA

## Abstract

Surgery patients in Japan undergo routine spirometry testing prior to general anesthesia. The use of a flow sensor during general anesthesia has recently become common. However, it is not certain whether the information derived from flow-volume curves is being adequately used for mechanical ventilation management during general anesthesia. So far, there have been no attempts to calculate airway resistance using flow-volume curves. Therefore, we performed a prospective, observational study to investigate the relationship between pre-anesthetic and intra-anesthetic airway resistance in patients scheduled for surgery under general anesthesia. We calculated pre-anesthetic and intra-anesthetic airway resistance in each patient, based on the slopes of flow-volume curves obtained prior to and during general anesthesia. We also calculated endotracheal tube resistance to correct the intra-anesthetic airway resistance values calculated. A total of 526 patients were included in the study, and 98 patients had a forced expiratory volume in the first second/forced vital capacity ratio of < 70%. Pre-anesthetic airway resistance was significantly higher in patients with airflow obstruction than in those without airflow obstruction (*p* < 0.001), whereas no significant difference in intra-anesthetic airway resistance was found between patients with and without airflow obstruction during mechanical ventilation (*p* = 0.48). Pre-anesthetic and intra-anesthetic airway resistance values were closer to each other in patients without airflow obstruction, with a mean difference < 1.0 cmH_2_O L^-1^s^-1^, than in those with airflow obstruction, although these respiratory parameters were significantly different (*p* < 0.001). Intra-anesthetic airway resistance was not related to the FEV1/FVC ratio, regardless of the degree to which the FEV1/FVC ratio reflected pre-anesthetic airway resistance. As compared with patients with airflow obstruction, the mean difference between pre-anesthetic and intra-anesthetic airway resistance was small in patients without airflow obstruction.

## Introduction

Spirometry is such a simple and reproducible test of lung function that it has become the gold standard method [1, 2]. Almost all surgery patients in Japan undergo spirometry testing as part of routine preoperative examinations, regardless of respiratory dysfunction. Despite the popularity of spirometry, however, it remains unclear to what extent the pre-anesthetic respiratory function parameters obtained from maximum expiratory flow-volume curves contribute to mechanical ventilation management during general anesthesia.

Airway resistance is generally measured using body plethysmography [3, 4] or the forced oscillation technique [5, 6], but these methods are not frequently used in clinical practice. However, it is feasible to calculate airway resistance using the slope of the descending limb of flow-volume curves, which is expressed as the reciprocal of the time constant [7]. Nevertheless, there have been few reported attempts to calculate airway resistance using flow-volume curves.

According to the concept established by Mead and his coworkers [8], maximal expiratory flow is independent of muscular effort as long as the effort is above certain levels. During forced expiration, the driving pressure is the alveolar pressure (Palv), which is the sum of the elastic recoil pressure (Pel) and pleural pressure (Ppl). Along the airway, there is a certain point called the equal pressure point (EPP), at which the airway pressure inside matches the Ppl. This point partitions the airway into the upstream segment where the transmural pressure Ptm is positive, and the downstream segment where Ptm is negative. The latter segment undergoes further dynamic compression with increasing Ppl, whereas the former remains unchanged. Maximal expiratory flow is thus a function of Pel and the resistance of the airway upstream from the EPP, Rus, can be represented as:

$$\dot{V}\text{max}=\frac{\text{Pel}}{\text{Rus}}$$ (1)

During mechanical ventilation, expiration is a passive process in which a single volume elastic element passively empties through a flow resistance, and the slope of the flow-volume curves is thus determined by the elastic recoil pressure of the lung and the flow resistance [9–11]. It then follows that the respiratory mechanics of mechanical ventilation would resemble a simple monoalveolar lung model in which Pel functions as the driving pressure for flow through the airways running from the alveoli to the EPP [12]. As far as the properties of the lungs and airways are also reflected in controlled conditions unaccompanied by forced expiration, such as mechanical ventilation during general anesthesia, it would then be worth comparing respiratory parameters obtained during forced expiration and mechanical ventilation.

We set out to test the hypothesis that pre-anesthetic airway resistance would theoretically be equal to intra-anesthetic airway resistance in patients with a normal forced expiratory volume in the first second (FEV1)/ forced vital capacity (FVC) ratio, but not in patients with an FEV1/FVC ratio < 70%. We thus conducted a prospective, observational study to investigate the relationship between pre-anesthetic and intra-anesthetic airway resistance in patients scheduled for surgery under general anesthesia, by calculating airway resistance based on flow-volume curves obtained prior to and during general anesthesia.

## Materials and methods

### Study population

This was a prospective, observational study conducted at the University of Tokyo Hospital, Tokyo (Japan) between April 5 and May 31, 2016. We included patients aged 15 years or older who were scheduled for non-cardiac surgery, including abdominal, gynecological, neurosurgical, orthopedic, otologic, ophthalmic, plastic, and dental operations under general anesthesia, and whose airways were managed by orotracheal intubation using a normal or spiral endotracheal tube. We excluded patients who underwent general anesthesia using a supraglottic airway device, who required one lung ventilation during thoracotomy, who were intubated with a double lumen endotracheal tube, or were intubated with a RAE endotracheal tube.

The institutional review board of the University of Tokyo approved the study design (IRB #11108). Informed consent to participate was deemed unnecessary, as the study was conducted during the course of normal clinical practice, and all patients scheduled for surgery at our institution undergo routine spirometry testing prior to general anesthesia, and use of a flow sensor during general anesthesia for optimizing ventilation monitoring is also routine practice.

### Calculation method

The recoil pressure of the element P is expressed as a single-value function of its volume V. Simultaneously, P is the driving pressure for the flow resistance through which the element empties, and the flow $\dot{V}$ is expressed as a single-value function of P. The chord conductance of the flow resistive element G and the chord elastance of the volume-elastic element E are defined as follows:

$$\text{G}=\frac{\dot{V}}{\text{P}}=\frac{1}{\text{R}}$$ (2)

$$\text{E}=\frac{\text{P}}{\text{V}}=\frac{1}{\text{C}}$$ (3)

where R is resistance and C is compliance, the reciprocals of conductance and elastance, respectively.

The descending limb of flow-volume curves after 25% expiration is effort-independent and almost linear, with flows falling to zero at the final volume [13, 14]. The slope of the descending limbs is calculated as:

$$\frac{\dot{V}}{\text{V}}=\frac{\dot{V}}{\text{P}}\times \frac{\text{P}}{\text{V}}=\text{GE}=\frac{1}{\text{CR}}$$ (4)

This equation defines the slope as the reciprocal of the time constant of the system, the product of airway resistance and lung compliance [12].

The pressure to overcome the elastic forces for a given tidal volume (TV) under static conditions, termed static lung compliance, reflects the elasticity of the lung parenchyma [15]. Utilization of parameters monitored during mechanical ventilation makes it possible to calculate static lung compliance, Cst, which is generally calculated as the reciprocal of elastance, by means of the following equation:

$$\text{Cst}=\frac{\text{TV}}{\text{Pplat}-\text{PEEP}}$$ (5)

where Pplat is the end-inspiratory plateau pressure, and PEEP is the positive end-expiratory pressure [16]. Airway resistance can theoretically be obtained based on the Eqs (4) and (5) above.

To guarantee the accuracy of evaluations of the characteristic traits of the respiratory system in each case, we calculated the slope of the effort-independent part of the descending limb. The expiratory flow rates at 50% and 25% of FVC ($\dot{V}50$ and $\dot{V}25$ respectively) were ascertained via spirometry, and the slope of the line passing through $\dot{V}50$ and $\dot{V}25$ was calculated as:

$$\frac{\dot{V}}{\text{V}}=\frac{\dot{V}50-\dot{V}25}{0.25\text{FVC}}$$ (6)

Using Eqs (5) and (6) above, we calculated pre-anesthetic airway resistance during forced expiration (expressed in this study as Rfe) in each case via the following equation:

$$\text{Rfe}=\frac{0.25\text{FVC}}{\text{Cst}\;\left(\dot{V}50-\dot{V}25\right)}$$ (7)

**Fig 1:**
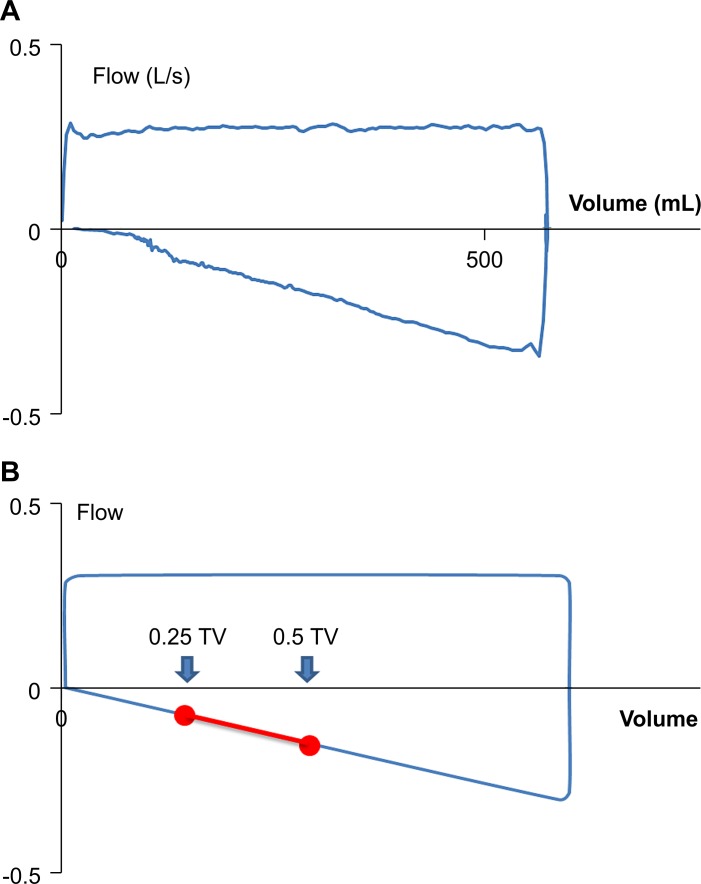
Flow-volume curves observed during mechanical ventilation. A: An example of a flow-volume curve recorded during general anesthesia. Its descending limb appears almost linear during mechanical ventilation. B: A schematic diagram of a flow-volume curve during mechanical ventilation. The interval connecting the points that correspond to the flow rates at 50% and 25% of tidal volume (expressed as $\dot{V}’50$ and $\dot{V}’25$, respectively) is displayed as a red line segment.

In a similar manner, we calculated intra-anesthetic airway resistance during mechanical ventilation (expressed in this study as Rmv) in each case. For a strict comparison of Rmv and Rfe, we evaluated the counterpart of the interval between $\dot{V}50$ and $\dot{V}25$ in the maximal expiratory flow-volume curve curves (Fig 1). We first identified the points that corresponded to the flow rates at 50% and 25% of TV (expressed as $\dot{V}\prime 50$ and $\dot{V}\prime 25$, respectively), and then calculated the slope of the line passing through $\dot{V}\prime 50$ and $\dot{V}\prime 25$.

**Fig 2:**
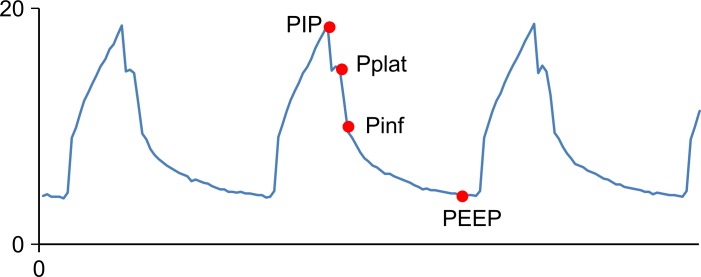
An example of the airway pressure profile of a volume-controlled breath. Closer observation of the change in airway pressure during the expiratory phase reveals that there is first a sharp drop from the end-inspiratory plateau pressure (Pplat) to an inflection point, which is eventually followed by a gradual decline to the positive end-expiratory pressure (PEEP) level, or zero.

Analysis of the airway pressure profile revealed a specific inflection point during the expiratory phase (Fig 2).

We estimated that this inflection point (expressed in this study as Pinf) would be equal to the outlet pressure at the beginning of expiration, and that the pressure difference between Pplat and Pinf would then correspond to the driving pressure ⊿P. On the presumption that the ratio of ⊿P to P is constant throughout expiration, the slope of the descending limb under mechanical ventilation was calculated as:

$$\frac{\dot{V}}{\text{V}}=\frac{⊿\text{P}}{\text{R}}\times \frac{1}{\text{PC}}=\frac{⊿\text{P}}{\text{P}}\times \frac{1}{\text{CR}}=k\times \frac{1}{\text{CR}}$$ (8)

where *k* is the ratio of ⊿P to the difference between Pplat and PEEP. Rmv was thus calculated in the same manner, as follows:

$$\text{Rmv}=k\times \frac{0.25\text{TV}}{\text{Cst}\;\left(\dot{V}\prime 50-\dot{V\prime }25\right)}$$ (9)

Patients undergoing general anesthesia were intubated in the supine position, but their body position was not necessarily constant during surgery. Therefore, we used flow-volume curves recorded between 3 and 4 min after orotracheal intubation and derived the average of the calculated Rmv and Cst values obtained during the 1-min period. This ensured that all patients were most likely in the supine position and under adequate muscle relaxation and excluded the effects of differences in surgeries, including pneumoperitoneum and changes in body position. We also confirmed that none of the patients had exhibited obvious signs of respiratory failure related to asthma or allergic disorders at least 1 month prior to general anesthesia.

### Measurement of endotracheal tube resistance

The calculated Rmv values were overestimated, and thus required correction by subtraction of endotracheal tube resistance (Rett). In attempting to study the relationship between pressure drop and flow, we measured Rett for each endotracheal tube with an inner diameter of 6.5–8.0 mm [17, 18].

We measured a pressure drop for each tube along its natural curvature; the distal end of each tube was open to the atmosphere, while compressed air was constantly delivered at flow rates of 0.2, 0.3, 0.4, 0.5, and 1.0 L/s. During mechanical ventilation, the maximum flow rate was set at 0.5 L/s, but we estimated that it would be appropriate to adopt a pressure drop similar to that observed at a flow rate between $\dot{V}\prime 50$ and $\dot{V}\prime 25$.

We then calculated Rett as pressure drop (measured in cmH_2_O) divided by flow (measured in L/s), and we obtained an average of five measured Rett values. The corrected Rmv value (expressed in this study as cRmv) was then calculated as:

cRmv=Rmv−Rett (10)

### Statistical analysis

We calculated that a sample size of 97 patients with an FEV1/FVC ratio < 70% would provide 90% power at an alpha level of 0.05, to detect a difference of 10 cmH_2_O L^-1^s^-1^ between pre-anesthetic and intra-anesthetic airway resistance [19]. Data are expressed as number and %, mean ± standard deviation (SD), or median and inter-quartile range (IQR).

Mean differences in the Cst, Rfe, and cRmv values between patients with a normal FEV1/FVC ratio and those with an FEV1/FVC ratio < 70% were assessed using the Satterthwaite *t*-test. Mean differences between the Rfe and cRmv values in each patient were assessed using the paired *t*-test, as these values were derived from the same patient. The relationships between FEV1/FVC ratio and Rfe and cRmv values were assessed using linear regression analysis. The relationship between Rfe and cRmv was assessed using linear regression analysis and Bland-Altman analysis. For non-parametric data, we used the Mann–Whitney test. A *p* value of less than 0.05 was considered statistically significant. All statistical analyses were performed using JMP Pro ver.10.0 (SAS Institute Japan Ltd, Tokyo, Japan) statistical software.

## Results

**Fig 3:**
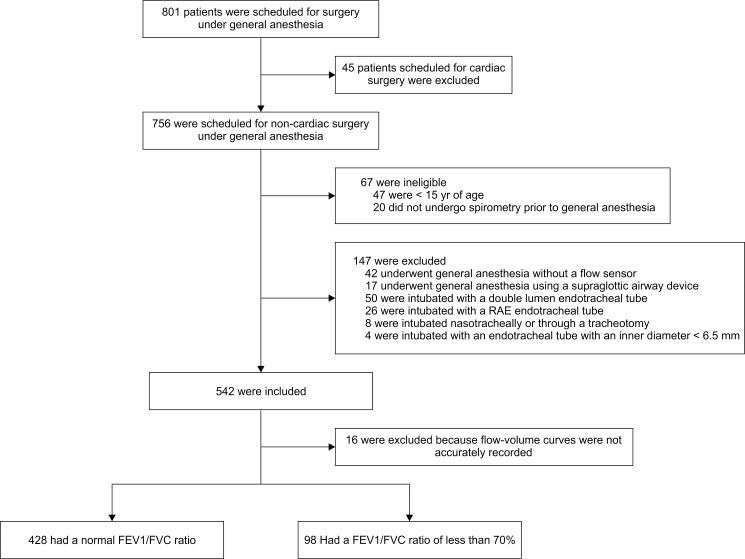
Assessment and follow-up of patients. A total of 801 consecutive patients were scheduled to undergo surgery under general anesthesia from April 5 to May 31, 2016. A total of 526 patients were included in the study analysis.

From April 5 to May 31, 2016, a total of 801 consecutive patients were scheduled for surgery under general anesthesia, of which 259 patients were deemed ineligible for inclusion in the study. The remaining 542 patients, who were orotracheally intubated using a normal or spiral endotracheal tube with an inner diameter ranging from 6.5 to 8.0 mm, were deemed eligible for initial inclusion in the study (Fig 3). Of these, we subsequently had to exclude 16 patients whose flow-volume curves were not accurately recorded during mechanical ventilation, creating difficulty in identifying the points corresponding to $\dot{V}\prime 50$ or $\dot{V}\prime 25$. Thus, 526 patients were ultimately included in the study analysis. For each of these patients, we calculated both Rfe and Rmv using Eqs (7) and (9) above, respectively.

**Fig 4:**
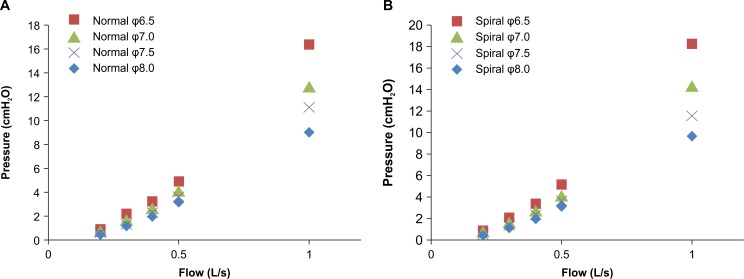
Dimensional representation of the pressure drop–flow relationship. We measured pressure drops for each tube, while compressed air was constantly delivered at a specific flow rate. There was a nearly linear relationship between pressure drop and flow when the flow rate was less than 0.5 L/s.

The relationship between pressure drop and flow approximated a linear relationship within a range of flow rates below 0.5 L/s (Fig 4). We adopted the pressure-drop value recorded at a flow rate of 0.2 L/s to calculate Rett, because this flow rate was nearly equal to that observed between $\dot{V}\prime 50$ and $\dot{V}\prime 25$ during mechanical ventilation. The mean Rett values for each endotracheal tube are listed in Table 1, and these mean values were used in Eq (10) above, to correct the Rmv value in each case.

**Table 1:** The calculated values of endotracheal tube resistance.

	Normal tube	Spiral tube
ID 6.5 mm (cmH_2_O L^-1^s^-1^)	4.51 ± 0.18	4.83 ± 0.07
ID 7.0 mm (cmH_2_O L^-1^s^-1^)	3.45 ± 0.03	3.93 ± 0.04
ID 7.5 mm (cmH_2_O L^-1^s^-1^)	2.90 ± 0.08	2.97 ± 0.07
ID 8.0 mm (cmH_2_O L^-1^s^-1^)	2.16 ± 0.07	2.47 ± 0.09

Data are presented as means ± standard deviation. ID = internal diameter.

In accordance with spirometric criteria for COPD, we used an FEV1/FVC ratio of 70% as a cut-off value, and in our study, 98 patients with an FEV1/FVC ratio < 70% were regarded as having airflow obstruction. The baseline characteristics of the patients are summarized in Table 2. The mean age was significantly higher in patients with an FEV1/FVC ratio < 70%, and more than 60% of these patients were male. Among the respiratory function parameters derived from spirometry, there were significant differences in FEV1, MMF, $\dot{V}50$, $\dot{V}25$, $\dot{V}50/\dot{V}25$, and $\dot{V}25/\text{Ht}$ between patients with a normal FEV1/FVC ratio and those with an FEV1/FVC ratio < 70%. Neither VC nor FVC differed significantly between the two groups.

**Table 2:** Baseline characteristics and respiratory function parameters obtained from spirometry.

	Patients with airflow limitation (N = 98)	Patients without airflow limitation (N = 428)	*p* value
Age (years)	68.98 ± 9.53	56.21 ± 16.03	< 0.001
Gender (male), n (%)	62 (63%)	180 (42%)	
Height (cm)	160.03 ± 9.72	160.72 ± 9.29	0.53
Body weight (kg)			
Actual	68.54 ± 73.23	60.34 ± 13.67	0.27
Predicted	55.30 ± 10.44	54.96 ± 9.93	0.77
Body mass index	26.34 ± 24.80	23.26 ± 4.37	0.22
ASA physical status			
1, n (%)	9 (9.2%)	151 (35%)	
2, n (%)	83 (85%)	258 (60%)	
3, n (%)	6 (6.1%)	19 (4.4%)	
FEV1 (L)	1.92 (1.58–2.34)	2.57 (2.11–3.05)	< 0.001
VC (L)	3.12 (2.56–3.64)	3.14 (2.66–3.76)	0.56
FVC (L)	3.08 (2.53–3.64)	3.13 (2.62–3.72)	0.52
MMF (L/s)	0.89 (0.61–1.16)	2.52 (1.79–3.31)	< 0.001
$\dot{V}50$ (L/s)	1.31 (0.97–1.78)	3.30 (2.53–4.14)	< 0.001
$\dot{V}25$ (L/s)	0.29 (0.21–0.42)	0.92 (0.60–1.39)	< 0.001
$\dot{V}50/\dot{V}25$	4.65 (3.65–5.50)	3.44 (2.72–4.36)	< 0.001
$\dot{V}25/\text{Ht}$ (L s^-1^m^-1^)	0.19 (0.14–0.27)	0.58 (0.39–0.85)	< 0.001

Data are presented as n (%), mean ± standard deviation, or median (interquartile range). The predicted body weight was calculated as follows: for men, 50 + 0.91 * (height in centimeters—152.4); and for women, 45.5 + 0.91 * (height in centimeters—152.4). The body mass index is the weight in kilograms divided by the square of the height in meters. ASA = American Society of Anesthesiologists. FEV1 = forced expiratory volume in one second. VC = vital capacity. FVC = forced vital capacity. MMF = maximum mid-expiratory flow rate. $\dot{V}50/\dot{V}25$ = the value of $\dot{V}50$ divided by that of $\dot{V}25$. $\dot{V}25/\text{Ht}$ = the value of $\dot{V}50$ divided by height.

Parameters relating to mechanical ventilation during general anesthesia, including modes of ventilation, TV, TV/predicted body weight, frequency, Pplat, PEEP, and Pinf, were similar between patients with a normal FEV1/FVC ratio and those with an FEV1/FVC ratio < 70% (Table 3). The Cst value was calculated in each case based on these parameters, and there was a statistically significant difference between the Cst values of the two groups (59.81 ± 14.4 mL/cmH_2_O in the normal group vs. 63.65 ± 15.9 mL/cmH_2_O in the < 70% group; *p* = 0.030), but the actual difference between the means was < 4.0 mL/cmH_2_O.

**Table 3:** Airway resistance, lung compliance, and mechanical ventilation-related parameters.

	Patients with airflow limitation (N = 98)	Patients without airflow limitation (N = 428)	*p* value
Cst (mL/cmH_2_O)	63.65 ± 15.9	59.81 ± 14.4	0.030
Rfe (cmH_2_O L^-1^s^-1^)	14.11 ± 7.69	6.49 ± 2.40	< 0.001
Rmv (cmH_2_O L^-1^s^-1^)	10.67 ± 3.17	10.62 ± 2.65	0.88
cRmv (cmH_2_O L^-1^s^-1^)	7.69 ± 3.14	7.44 ± 2.60	0.48
Ventilation mode			
VCV	77 (79%)	318 (74%)	
PCV	21 (21%)	110 (26%)	
TV (mL)	0.55 (0.48–0.61)	0.52 (0.46–0.59)	0.056
TV/PBW (mL/kg)	9.71 (8.64–11.52)	9.58 (8.31–10.97)	0.29
Frequency (/min)	10 (10–10)	10 (10–10)	0.30
PIP (cmH_2_O)	14.54 (12.79–16.47)	14.25 (12.62–16.25)	0.69
Pplat (cmH_2_O)	12.54 (10.61–14.13)	12.27 (10.63–14.47)	0.97
Pinf (cmH_2_O)	7.83 (5.27–9.34)	7.44 (5.36–8.99)	0.51
PEEP (cmH_2_O)	3.96 (1.84–5.02)	3.22 (1.71–4.98)	0.23
Tube type			
Normal, n (%)	74 (76%)	333 (78%)	
Spiral, n (%)	24 (24%)	95 (22%)	
Tube size (ID)			
6.5 mm, n (%)	3 (3.1%)	20 (4.7%)	
7.0 mm, n (%)	32 (33)	219 (51%)	
7.5 mm, n (%)	35 (36)	101 (24%)	
8.0 mm, n (%)	28 (29%)	88 (21%)	

Data are presented as n (%), mean ± standard deviation, or median (interquartile range). Cst = static lung compliance. Rfe = airway resistance under forced expiration. Rmv = airway resistance under mechanical ventilation. cRmv = corrected airway resistance under mechanical ventilation. VCV = volume-controlled ventilation. PCV = pressure-controlled ventilation. TV = tidal volume. Pplat = plateau pressure. Pinf = the airway pressure that corresponds to an inflection point during the expiratory phase.

PEEP = positive end-expiratory pressure.

**Fig 5:**
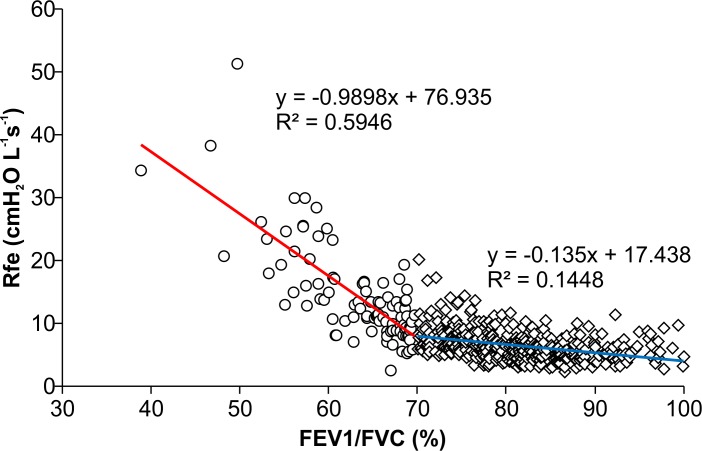
Scatter plots showing the relationship between the FEV1/FVC ratio and Rfe. Rfe increased when the FEV1/FVC ratio was < 70%. The determination coefficient was 0.5946 in patients with an FEV1/FVC ratio < 70%, and 0.1448 in those with a normal FEV1/FVC ratio.

**Fig 6:**
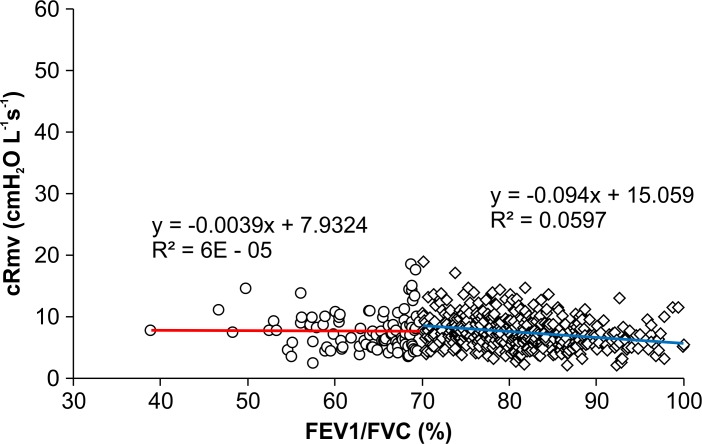
Scatter plots showing the relationship between the FEV1/FVC ratio and cRmv. cRmv decreased as compared with Rfe, and there was no significant difference between patients with a normal FEV1/FVC ratio and those with an FEV1/FVC ratio < 70%.

Table 3 includes the results for pre-anesthetic and intra-anesthetic airway resistance calculated based on the slope of flow-volume curves. Figs 5 and 6 show the Rfe and cRmv distributions according to the FEV1/FVC ratio. The mean value of Rfe was significantly higher in the group with an FEV1/FVC ratio < 70% than in the normal FEV1/FVC ratio group (14.11 ± 7.69 vs. 6.49 ± 2.40 cmH_2_O L^-1^s^-1^; *p* < 0.001). In the former group, an R-square value of 0.5946 was obtained between the FEV1/FVC ratio and the Rfe value, whereas in the latter group, the R-square value was 0.1448 (Fig 5). There was no significant difference in cRmv between the two groups (7.69 ± 3.14 cmH_2_O L^-1^s^-1^ in the < 70% group vs. 7.44 ± 2.60 cmH_2_O L^-1^s^-1^ in the normal group; *p* = 0.48), and the R-square value was nearly zero in each group (Fig 6).

**Fig 7:**
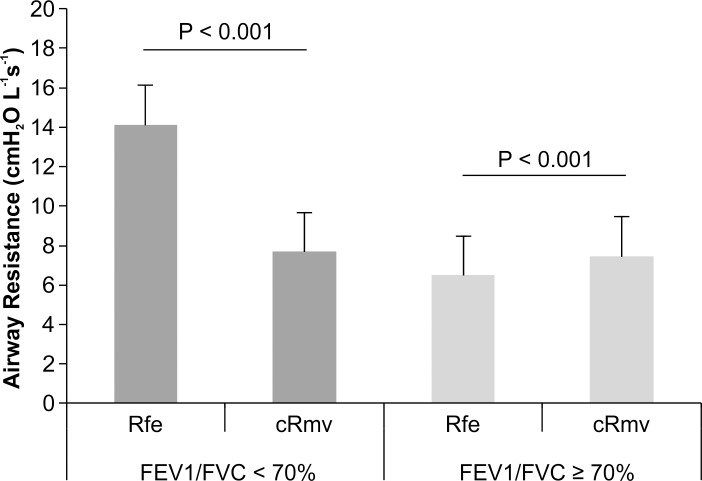
Visual representation of the mean difference between the calculated values of Rfe and cRmv. The mean difference between Rfe and cRmv was larger in patients with an FEV1/FVC ratio < 70% than in those with a normal FEV1/FVC ratio, but there was a significant difference between Rfe and cRmv in both patient groups.

**Fig 8:**
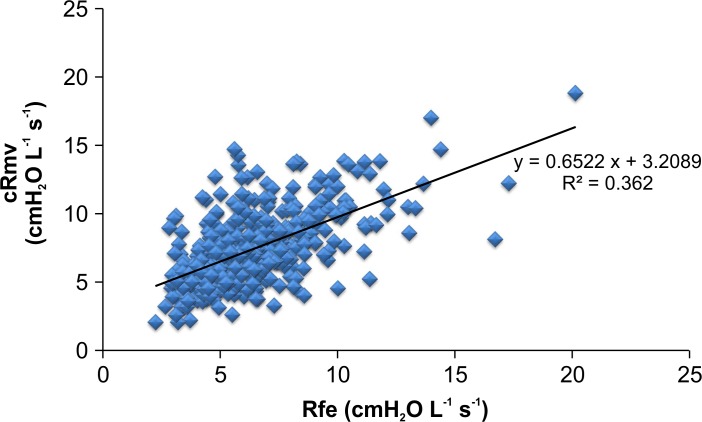
Scatter plots showing the relationship between Rfe and cRmv in patients with a normal FEV1/FVC ratio. To a certain extent, Rfe was associated with cRmv, with a determination coefficient of 0.362 in patients with a normal FEV1/FVC ratio.

**Fig 9:**
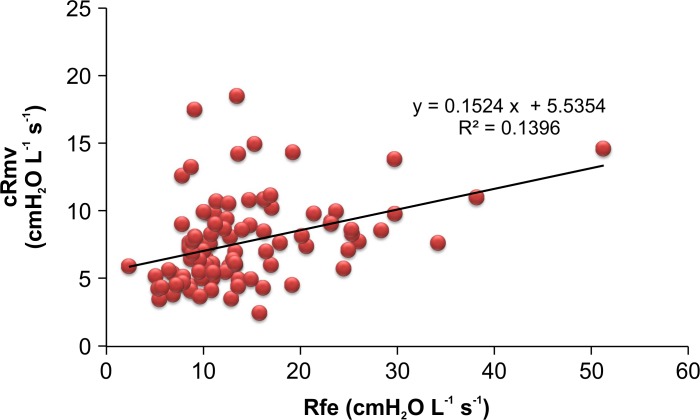
Scatter plots showing the relationship between Rfe and cRmv in patients with an FEV1/FVC ratio < 70%. The determination coefficient was 0.1396 in patients with an FEV1/FVC ratio < 70%, showing that Rfe was less associated with cRmv in these patients than in those with a normal FEV1/FVC ratio.

**Fig 10:**
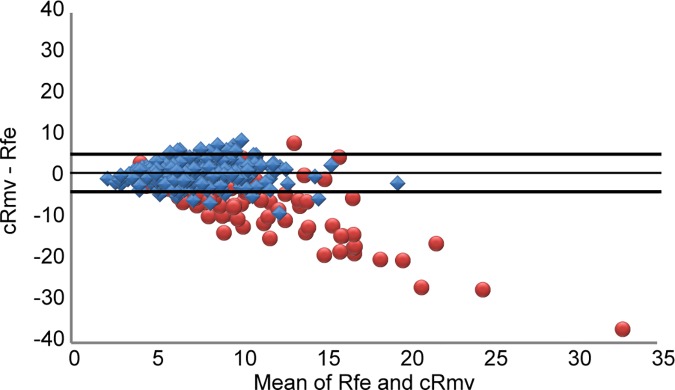
Bland-Altman plot of differences in airway resistance (cRmv–Rfe) against the mean of Rfe and cRmv. Blue dots represent patients with a normal FEV1/FVC ratio, and the middle, top, and bottom black lines represent the mean difference, and boundaries of the 95% limits of agreement, respectively. Red dots representing patients with an FEV1/FVC ratio < 70% were also added for contrast.

In patients with a normal FEV1/FVC ratio, the difference between Rfe and cRmv was less than 1.0 cmH_2_O L^-1^s^-1^ (Table 3), but this difference was statistically significant (7.44 ± 2.60 vs. 6.49 ± 2.40 cmH_2_O L^-1^s^-1^, respectively; *p* < 0.001) (Fig 7). In patients with an FEV1/FVC ratio < 70%, the cRmv value was significantly lower than the Rfe value (7.69 ± 3.14 vs. 14.11 ± 7.69 cmH_2_O L^-1^s^-1^; *p* < 0.001). The R-square value was 0.3620 in patients with a normal FEV1/FVC ratio, and 0.1396 in those with an FEV1/FVC ratio < 70% (Figs 8 and 9). According to the Bland-Altman analysis, the 95% limits of agreement between Rfe and cRmv were 5.44 and -3.53 cmH_2_O L^-1^s^-1^, with a mean difference (cRmv–Rfe) of 0.95 cmH_2_O L^-1^s^-1^ in patients with a normal FEV1/FVC ratio (Fig 10).

## Discussion

In this prospective, observational study, Rfe and cRmv values were more similar among patients without airflow obstruction than among those with airflow obstruction whose FEV1/FVC ratio was < 70%, although these respiratory parameters remained significantly different. Mean Rfe was significantly higher in patients with airflow obstruction than in those without airflow obstruction, whereas there was no significant difference in cRmv between patients with and without airflow obstruction.

In this study, we calculated the slopes of expiratory flow-volume curves for the purpose of comparing pre-anesthetic and intra-anesthetic airway resistance. The reason for the validity of this comparison, despite the difference in measurement conditions, is that we adopted the effort-independent part of the descending limb of flow-volume curves, where flow depends on the elastic recoil pressure of the lung and the flow resistance [8, 12–14]. The finding that the Rfe value was rather close to the cRmv value without the presence of pre-anesthetic airflow obstruction may suggest that the properties of the lungs and airways measured by spirometry testing is reflected in the respiratory mechanics of mechanical ventilation during general anesthesia. The importance of our study therefore lies in the implication that pre-anesthetic and intra-anesthetic airway resistance calculated based on flow-volume curves obtained prior to and during general anesthesia are comparable, regardless of the difference in their origins.

Through our study, we managed to calculate airway resistance by maximizing the utilization of flow-volume curves, instead of using a special devices or approaches, such as body plethysmography or the forced oscillation technique. In a similar study examining the correlations between flow-volume curves prior to and during general anesthesia, the ratio of the slope between $\dot{V}75$ and $\dot{V}50$ to that between $\dot{V}25$ and end-expiration was used to evaluate the slopes of flow-volume curves [20]. In our study, however, because greater emphasis was placed on the accuracy of evaluation of the characteristic traits of the respiratory system, we adopted the slopes of the effort-independent part of the descending limb with their counterparts in flow-volume curves during mechanical ventilation, i.e., the slopes of the lines passing through $\dot{V}50$ and $\dot{V}25$, and through $\dot{V}’50$ and $\dot{V}’25$, respectively. It was this calculation method based on respiratory mechanics that made it possible to perform the study by simply using information readily available in normal clinical practice.

The FEV1/FVC ratio, one of the most important parameters obtained from spirometry, was closely associated with Rfe. Within the range of a normal FEV1/FVC ratio, Rfe was close to the mean value, exhibiting diminished variance. In contrast, however, Rfe was increased in conjunction with a reduced FEV1/FVC ratio of < 70%. This finding, in which two obviously different tendencies were observed, depending on whether the FEV1/FVC ratio was below or above 70%, is consistent with the fact that the use of spirometry has been recommended as the gold standard for the detection of airflow obstruction [1, 2, 21, 22].

In contrast with the extent to which the FEV1/FVC ratio was reflective of Rfe, no significant difference was found in cRmv between patients with a normal FEV1/FVC ratio and those with an FEV1/FVC ratio < 70%. In our study, there was no increase in airway resistance during mechanical ventilation in patients with airflow obstruction, whereas some studies have reported markedly increased airway resistance in patients with chronic obstructive pulmonary disease (COPD) who were mechanically ventilated in the intensive care unit [23, 24]. This could probably be attributed to the difference in the severity of COPD, as the patients included in these studies had more severe respiratory function, compared with those in our study, and required mechanical ventilation due to acute exacerbation of COPD.

The collapsibility of the site into which an endotracheal tube is inserted might have been related to the increase in Rfe in patients with an FEV1/FVC ratio < 70%. It is widely considered that the increased airway resistance in COPD results from pathology of the small airways [25–27]. The small airways are nevertheless referred to as “the silent zone of the lung,” as their contribution to the total airway resistance is rather small [28]. On the other hand, the chief site of airway resistance lies in the larger and medium-sized airways [29]. There is also a clinical syndrome involving the collapsibility of the central airways, known as expiratory central airway collapse (ECAC) that is characterized by airflow obstruction due to excessive narrowing of the central airway lumen during expiration [30]. The presence of cartilaginous rings that encase the central airway circumferentially, except for the posterior area, which is occupied by membranous structures, protects the large central airways from dynamic compression and collapse [30]. The membranous part of the central airways often bows inwards during expiration in ECAC [31, 32], but it is not clarified whether dynamic narrowing of the central airways is responsible for increase in airway resistance during forced expiration.

The effects of the airway resistance associated with the anesthetic agents used for the induction and maintenance of general anesthesia should be taken into account. Most inhaled anesthetics are known to function as dose-dependent bronchodilators [33, 34], and intravenous anesthetics, such as propofol can also have a similar effect [35], although it is not as marked as that of inhaled anesthetics. Airway resistance may be expected to decrease during general anesthesia, but cRmv was not lower than Rfe in patients without airway obstruction. Moreover, the observation that a substantial increase in pre-anesthetic airway resistance in patients with airway obstruction was completely absent during mechanical ventilation can hardly be attributed solely to the effects of anesthetic agents. It was recently reported that some inhaled anesthetics failed to produce bronchodilation at 1.0 and 1.5 minimum alveolar concentration [36], and anesthetic agents would have produced limited effects in our study.

There are several limitations to our study. First, we had to employ the Cst values derived from intra-anesthetic parameters of mechanical ventilation to calculate pre-anesthetic and intra-anesthetic airway resistance in each case. Our intention was to calculate lung compliance without employing an invasive test, such as the esophageal balloon technique. To minimize the effects of the differences in the surgery type and the intraoperative body position, we used parameters recorded shortly after orotracheal intubation, while all the patients were anesthetized and in the supine position. There were still statistically significant differences between patients with and without airflow obstruction, although the mean differences were rather small. Second, differences in the types and doses of anesthetic agents used were not adequately evaluated. Notably, however, the degree of dose-dependent bronchodilation caused by inhaled anesthetics would have been rather inconsequential, considering that airway resistance during general anesthesia was, contrary to expectations, not lower than that measured during forced expiration in patients with normal respiratory function. Finally, it should possibly be taken into account that, in our study, patients with an FEV1/FVC ratio < 70% were not necessarily diagnosed as having COPD. Spirometry plays an important role in detecting airflow obstruction, but it should be understood that there are also limitations to the spirometric criteria for COPD [37].

In conclusion, our study provided insight into the relationship between pre-anesthetic and intra-anesthetic airway resistance calculated on the basis of flow-volume curves obtained prior to and during general anesthesia. There was a significant difference between these two respiratory parameters, but the observation that pre-anesthetic airway resistance to some extent approximated intra-anesthetic airway resistance in the absence of airflow obstruction may imply that flow-volume curves can potentially be used as the source of respiratory parameters during mechanical ventilation. In our study, no increase in intra-anesthetic airway resistance was found in patients with airflow obstruction. Further investigation is required to determine whether increase in airway resistance is observed under controlled conditions in individuals with more severe respiratory dysfunction, which may eventually provide a clue to better respiratory management of these patients.

## Supporting information

S1 Appendix The data set. The data provided in this article are available within this file.
(XLSX)
